# Temporal Feature Extraction from DCE-MRI to Identify Poorly Perfused Subvolumes of Tumors Related to Outcomes of Radiation Therapy in Head and Neck Cancer

**DOI:** 10.18383/j.tom.2016.00199

**Published:** 2016-12

**Authors:** Daekeun You, Madhava Aryal, Stuart E. Samuels, Avraham Eisbruch, Yue Cao

**Affiliations:** 1 Department of Radiation Oncology, University of Michigan, Ann Arbor, Michigan;; 2 Department of Radiation Oncology, University of Miami, Miami, Florida; and; 3 Department of Radiation Oncology, Radiology, and Biomedical Engineering, University of Michigan, Ann Arbor, Michigan

**Keywords:** DCE-MRI, tumor subvolumes, therapy assessment, feature extraction, discrete WT, SVM

## Abstract

This study aimed to develop an automated model to extract temporal features from DCE-MRI in head-and-neck (HN) cancers to localize significant tumor subvolumes having low blood volume (LBV) for predicting local and regional failure after chemoradiation therapy. Temporal features were extracted from time-intensity curves to build classification model for differentiating voxels with LBV from those with high BV. Support vector machine (SVM) classification was trained on the extracted features for voxel classification. Subvolumes with LBV were then assembled from the classified voxels with LBV. The model was trained and validated on independent datasets created from 456 873 DCE curves. The resultant subvolumes were compared to ones derived by a 2-step method via pharmacokinetic modeling of blood volume, and evaluated for classification accuracy and volumetric similarity by DSC. The proposed model achieved an average voxel-level classification accuracy and DSC of 82% and 0.72, respectively. Also, the model showed tolerance on different acquisition parameters of DCE-MRI. The model could be directly used for outcome prediction and therapy assessment in radiation therapy of HN cancers, or even supporting boost target definition in adaptive clinical trials with further validation. The model is fully automatable, extendable, and scalable to extract temporal features of DCE-MRI in other tumors.

## Introduction

Dynamic contrast-enhanced (DCE) magnetic resonance imaging (MRI) (DCE-MRI; Table 1) has been widely explored and applied in clinical studies for diagnosis, treatment planning, and monitoring therapy response of diseases (1), particularly in cancers (2, 3). The T1-weighted DCE images are acquired during an intravenous bolus injection of a gadolinium-based contrast agent. The conventional analysis of DCE data is to quantify kinetic parameters such as perfusion, microvascular volume, vessel permeability, and volume of the extravascular extracellular space by fitting the data to a pharmacokinetic (PK) model (eg, Tofts model) (1, 2, 4). To further use this technique for cancer prognosis and therapy monitoring, a 2-step analysis is often applied, in which a metric(s) is extracted from physiological parametric maps and modeled for prediction of a clinical endpoint of interest. One of the advantages of PK modeling is that the estimated kinetic parameters have a physiological basis to an extent, and thereby, it is possible to compare with parameters across centers (1). However, these 2-step processes are time-consuming for processing a large amount of patient data and supporting real-time clinical decision-making.

**Table 1.** Frequently Used Abbreviations

Abbreviation	Description
AIF	Arterial Input Function
BV	Blood Volume
c-HBV	Class of High Blood Volume
c-LBV	Class of Low Blood Volume
CRT	Chemoradiation Therapy
DCE-MRI	Dynamic Contrast-Enhanced Magnetic Resonance Imaging
DSC	Dice Similarity Coefficient
GTV	Gross Tumor Volume
HBV	High Blood Volume
LBV	Low Blood Volume
PCA	Principal Component Analysis
PK	Pharmacokinetic
s-HBV	Subvolume of High Blood Volume
s-LBV	Subvolume of Low Blood Volume
SVM	Support Vector Machine
WT	Wavelet Transform

An emerging approach, data-driven machine learning, has the potential to process a large quantity of image data, extract “features” beyond expert's eyes, and create predictive models, which is termed *Radiomics* (5–7). The “features” extracted from the images range from textural features to intensity variation to tumor morphology. When applying this concept to the 4-dimensional DCE images, temporal “features” can be extracted, learned, and modeled in a fully automated process. The new data-driven machine learning approaches attempt to extract temporal “features” beyond the empirical parameters, for example, an area under the time-intensity curve, time-to-peak, peak enhancement, and wash-in and wash-out slopes, which are quantified by the conventional PK model-free approaches (1, 2).

In this study, we proposed a data-driven machine learning approach for extracting significant tumor subvolumes as an automatic supportive tool for radiotherapy assessment. A previous study (8) has shown that large poorly perfused subvolumes of either primary or nodal head and neck (HN) cancers before treatment and persisting during the early course of chemoradiation therapy (CRT) have the potential for predicting local and regional failure, and could be candidates for local dose intensification. We aimed to develop an automated and scalable model to identify subvolumes of the tumor with low blood volume (LBV) by extracting temporal contrast-enhanced features of DCE-MRI in HN cancer for predicting local and regional failure. In particular, we tested discrete wavelet transform (WT) (9) and principal component analysis (PCA) to characterize “features” in the DCE curves of HN cancers. Support vector machine (SVM) classifiers were trained regarding the temporal features to separate tumor voxels with LBV from those with high blood volume (HBV). We validated our method at voxel and patient levels using independent data sets. In addition, we evaluated whether our model without retraining could be applied to the DCE data acquired by a different pulse sequence on a different vendor scanner. Our results showed that the new approach reached high voxel classification accuracy of the tumor with LBV, which has the potential to automatically analyze the DCE data and create significant tumor behavior metrics for supporting adaptive RT in advanced HN cancers. The methodology can be fully automated and scalable to process a large DCE data set, and is also applicable to data sets obtained by different acquisition settings, such as different acquisition parameters or scanners.

## Material

### Patients and Image Data

The data used in this study were from 38 patients (female, 10; male, 28; median age, 58 years) who had HN cancers (T stage of 1–4 and N stage of 0–3) and were treated by CRT. The study was approved by the institutional review board of the University of Michigan. All patients underwent 3-dimensional DCE image scanning on a 3 T scanner (Skyra, Siemens Medical Systems, Erlangen, Germany) before CRT, using a TWIST pulse sequence in the sagittal plane with the following parameters: voxel size = 1.56 × 1.56 × 1.5 mm^3^; echo time/relaxation time = 0.9/2.58 milliseconds; temporal resolution = ∼3 seconds; and dynamic image volumes = 60.

**Figure 1.**
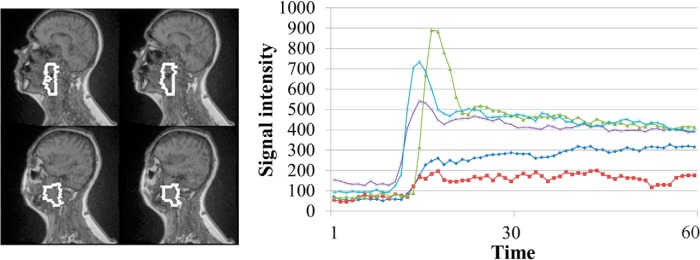
Example raw dynamic contrast-enhanced (DCE) curves from a primary gross tumor volume (white contours) in a patient. Note: Variations in the DCE curves.

Training and validation data sets for the proposed models consisted of 456 873 signal-intensity time-curves of DCE-MRI from 45 gross tumor volumes (GTVs), including primary and nodal tumors, of the first 32 patients (examples shown in Figure 1). The GTVs were delineated on magnetic resonance images by radiation oncologists. This data set was divided into training and validation subsets consisting of 70% and 30% of the curves, respectively. Each curve was labeled as either a class of LBV (c-LBV) or class of HBV (c-HBV) according to the blood volume (BV) of the corresponding voxel for classification model training and validation. The BV maps were obtained by fitting the same DCE-MRI data to a PK model (2-compartment Tofts model) (8, 10). The labeled DCE curves of each class were randomly selected to the training and validation data sets. To further validate the performance of the method at the patient level, the DCE data from the next 6 patients were used to create the subvolumes with LBV using the proposed method and compared with the data created using the 2-step method (8).

## Machine Learning Models

**Figure 2.**
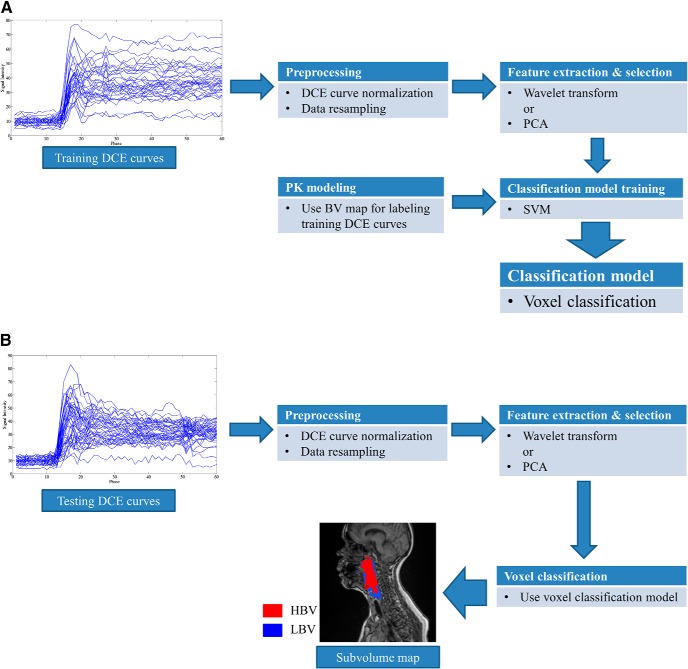
Block diagram of our proposed method. Classification model training phase (A). Usage (Validation) phase (B).

Our proposed framework is illustrated in Figure 2, and it consists of development and usage phases. The development phase (Figure 2A) includes training and validation steps, which use independent data sets. After validation, to use the model, a new patient's data can be rapidly processed through the workflow, without classification model training or PK modeling (Figure 2B). For the training process, the BV map derived from a PK model is only for labeling the voxels with LBV. Details of each step are discussed in this section.

### Preprocessing

The dynamic curve at each voxel represents the temporal changes in signal intensities after contrast injection. The curves need to be preprocessed to remove variations in intensity magnitude and onset time of contrast enhancement because of individual hemodynamics and various acquisition protocols. A signal intensity change at time *t*, Δ*S*(*t*), after contrast injection compared with a baseline intensity, (*S*_0_), is computed, and then normalized to the peak of the arterial input function (*AIF*_*max*_) of the patient as shown in the study by Farjam et al. (11) and using the following equation:

(1) $$\begin{array}{c} \Delta \text{S}\left(\text{t}\right)=\frac{\text{S}\left(\text{t}\right)-{\text{S}}_{0}}{{\text{S}}_{0}}\\ \Delta {\text{S}}_{\text{N}}\left(\text{t}\right)=\Delta \text{S}\left(\text{t}\right)\frac{1}{{\text{AIF}}_{\max}} \end{array}$$

where *S*(*t*) is the signal intensity of a DCE curve at time *t*, Δ*S*_*N*_(*t*) is normalized *S*(*t*), and *AIF*_*max*_ is the peak enhancement in Δ*S*(*t*) of the AIF. The AIF was determined by thresholding the intensity changes in a region of interest in a large artery, for example, *carotid artery* in this study.

For temporal feature extraction, a time-intensity series dyadic in length is required. In total, 32 time points with ∼3-second temporal resolution were selected starting from the onset time of the AIF (*AIF*_*onset*_) of each patient, so as to include the most significant intensity changes in the initial enhancement and the peak and to exclude the precontrast points and the extended wash-out period where minor intensity changes, in general, are observed. The use of the *AIF*_*onset*_ of each patient effectively removes patient variations in the onset time of enhancement in the time-intensity curves.

### Feature Extraction

To extract temporal features from the DCE curves and thereby differentiate the voxels with LBV from those with HBV, WT and PCA are applied to the preprocessed (normalized) DCE data to extract 2 different sets of features tested in 2 different models.

#### Wavelet Transform.

**Figure 3.**
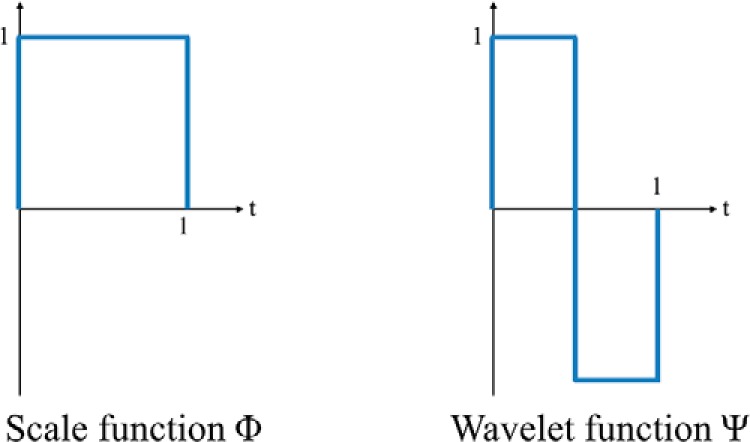
Discrete Haar wavelet functions.

WT is a multiresolution analysis that decomposes a signal into different frequency components in different scales (9). One-dimensional discrete Haar WT (HWT) is applied to characterize temporal frequency information in the DCE curves. The Haar WT computes sums and differences of pairs of temporal data points iteratively, and it stores the difference as a detail coefficient and passes the sum for the next iteration. The sum and difference are equivalent to low- and high-pass filters, respectively. The sum preserves low-frequency components and removes high-frequency fluctuations from the signal, whereas the difference captures high-frequency information. The low- and high-pass filtering can be performed by applying a scale (Φ) and WT (Ψ) functions (shown in Figure 3) to an input signal. The WT coefficients can be arranged as W = [A_J_, D_J_, D_J−1_, . . . , D_2_, D_1_], where *A*_*J*_ is the approximation coefficient and *D*_*j*_ represents detail coefficients at the *j^th^* scale level and is denoted as follows:

(2) $${\text{D}}_{\text{j}}=\{{\text{d}}_{\text{j}}^{\text{i}}|1\leq \text{i}\leq 2^{\left(\text{J}-\text{j}\right)}\}$$

where *d_j_^i^* is an individual coefficient and *J* is the maximum scale level and related to the number (N) of time points of the signal curve by N = 2^*J*^. It is worthwhile to point out that *A*_*J*_ is proportional to the area under the curve of the time-course (ie, *AUC*/*2^J^*). A full HWT is performed to yield 2^*J*^ coefficients including 1 approximation coefficient and (*2^J^* − 1) detail coefficients.

#### Principal Component Analysis.

PCA (12) is another method to extract features from the DCE-MRI data set. PCA is performed on the training data set to obtain principal components (PCs), which transform the DCE curves into the new feature space defined by the PCs. In our application, 32 PCs were initially obtained from the preprocessed DCE curves in the training data set, and projection coefficients of a new temporal curve *x*, that is, a representation of *x* in the PC space, were computed and used as features representing the curve *x*.

#### Feature Selection.

Feature (attribute) selection is an essential step in machine learning and data mining to reduce the dimensionality of initial states of features (13). Features can be easily composed of several tens or hundreds of attributes. A large dimensionality can hinder rapid model development and processing of a large data set. Both WT and PCA features in our application have only 32 attributes; however, the training set contains >300,000 DCE curves (samples) and, therefore, the model training can consume significant time without feature selection.

Among the major approaches in the literature (13), a *filter* method is used primarily because of its scalability and fast selection. The *wrapper* method can interact with the classifiers and model feature dependencies; however, it has a risk of over fitting and is slow and very computationally expensive compared with the filter method, and therefore, it may not be appropriate for a large training set.

The filter method was applied only to WT features. For PCA features, we selected the first several PCs with the largest eigenvalues, which have been frequently performed for feature selection or dimensionality reduction in PCA-based approaches (11, 14, 15).

### Voxel Classification

#### Voxel Labeling.

Voxel classification is performed using SVM (16). SVM is a supervised learning approach requiring labels of training samples (ie, DCE curves). The voxels in both training and validation data sets are labeled “0” for LBV and “1” for HBV based on the BV of each voxel [computed from Tofts model as described previously (8)]. A BV threshold of 7.6% that was previously established in Wang et al.'s study (8) was applied to determine the voxel labels.

#### Support Vector Machine.

SVM is a machine learning algorithm for finding a hyperplane splitting the labeled training samples into 2 classes (binary classification). The hyperplane is found to have a maximum distance (margin) from it to the nearest training samples on each side, known as a maximum-margin hyperplane. SVMs have several training parameters that can be adjusted during the training to build a better classification model. Equations 3 and 4 show the SVM objective function with a soft margin that allows for an analytical treatment of learning with errors (misclassification) on the training set as follows:

(3) $$\underset{\text{w},\text{b},\text{ξ}}{\text{argmin}}(\frac{1}{2}{\left\|\text{w}\right\|}^{2}+\text{C}\overset{\text{N}}{\underset{\text{i}=1}{\sum }}{\text{ξ}}_{i})$$

subject to the following equation:

(4) $$\begin{array}{c} y_{i}(w^{T}x_{i}+\text{b})\geq 1-\xi_{i},\\ \xi_{i}\geq 0, \end{array}\;\;\;\;\;\;\;\;\;\text{i}=1,. . .,\text{N}$$

where *w* and *b* define a hyperplane (decision surface), *N* is the number of training samples, and *y*_*i*_ is a ground truth label of the *i^th^* training sample **x**_*i*_. In the equation, ξ_*i*_ is a non-negative slack variable that measures the degree of misclassification of the **x**_*i*_ (eg, distance from **x**_*i*_ to the hyperplane). The constant *C*, a regularization parameter, is one of the SVM training parameters, which controls the cost of misclassification on the training data (tradeoff between error and margin). With a sufficiently small *C*, training errors (ie, sum of ξ_*i*_) can be ignored and it allows a large margin. A sufficiently large *C*, in contrast, makes training errors hard to be ignored and therefore results in a narrow margin, which could result in an over-fitted model to the training samples and take longer training time.

For training data with no linear hyperplane available in the original input space, a kernel-based SVM is used to implicitly transform the original input space into a higher-dimensional feature space, where a linear separating hyperplane is applicable. We used a radial basis function kernel with a trainable kernel parameter γ as follows:

(5) $$K(x_{i},\;x_{j})=\exp(-\gamma {\left|x_{i}-x_{j}\right|}^{2}),\gamma >0$$

where ***x***_*i*_ and ***x***_*j*_ are 2 sample vectors in the original input space. A sufficiently large γ may result in an overfitting and therefore poor generalization of the model.

#### Voxel Classification.

**Figure 4.**
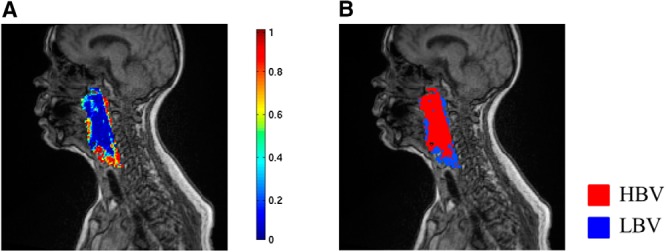
Class probability map and subvolume map. Posterior probability of c-LBV (A). Subvolume map derived from the map (A) by 0.5 threshold (B).

After training, the classifier assigns each voxel to either c-LBV or not, based upon the posterior class probability of the voxel either >0.5 or not as a final assignment of each instance. Then, a tumor subvolume with LBV is assembled from a group of voxels assigned to c-LBV by the classifier. Figure 4 shows the posterior probability map of c-LBV (the probability of a voxel being classified as LBV), and the corresponding subvolume map derived from the probability map in Figure 4A with a threshold of 0.5.

### Software Packages

We used Weka data mining software package (17) for feature selection from the WT features. A filter method based on correlation-based feature subset selection (*CfsSubsetEval* in Weka) was used with different search methods, such as *Best-first*, *Genetic*, and *Greedy* search, and the features selected from each search method were compared for determining a final feature set.

LIBSVM (18), a library for support vector classification, was used to train SVM classifiers. We trained radial basis function kernel SVMs with a probability estimation option to obtain both an output class label and a posterior class probability of each voxel. The SVM parameters *C* and γ were empirically selected as 200 and 0.2, respectively.

## Validation

Validations were performed on data sets independent of the training data set at voxel and patient levels. The voxel-level validation used 30% of the DCE curves randomly selected from 456 873 curves of the first 32 patients. The patient-level validation used 6 new patients. In addition, a preliminary test was performed for the model sensitivity to the different DCE acquisition using the data from 2 patients who were scanned using a 3 T Philips scanner (Intera Achieva, Philips Medical Systems, Best, The Netherlands) with different pulse sequence and parameters.

### Metrics

Outcomes from our method were voxel classification and tumor subvolumes derived from the classification result. We had 2 performance measurements, namely, *accuracy* and the *Dice similarity coefficient* (DSC). Voxel classification accuracy was defined as follows:

(6) $$\text{Accuracy}(\%)=\;\frac{\#\text{of correctly classified voxels}}{\#\text{of total voxels}}$$

The DSC was used to compare the resulting subvolumes from the classification with a ground truth (defined by the BV threshold) to evaluate spatial overlap accuracy between the 2 segmentations. DSC was computed as follows:

(7) $$\text{DSC}(\text{G, R})=\frac{2|\text{G}\cap \text{R}|}{\left|\text{G}|+|\text{R}\right|}$$

where |.| denotes the total number of voxels in a segmentation; *G* and *R* denote ground truth and classification result, respectively; and ∩ is the intersection between the 2 segmentations. The 6 patients' data, which were not used for model training and testing, were mainly used for DSC evaluation.

## Results

### Wavelet Feature Extraction and Selection

**Figure 5.**
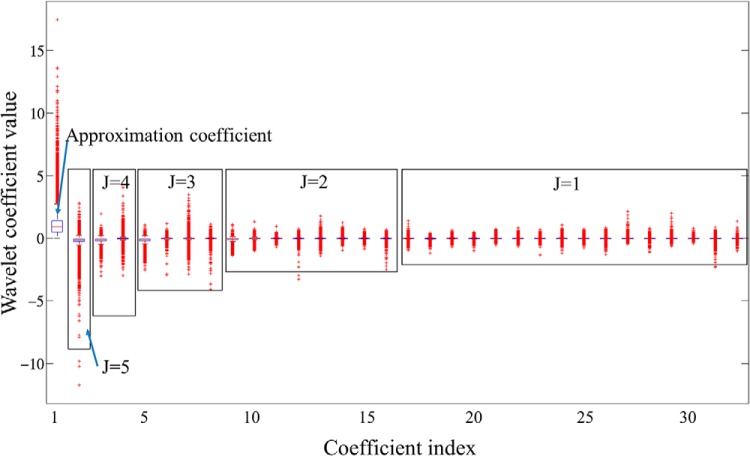
Box plot of coefficient variations obtained from the training curves. The first coefficient is A_5_ (approximation coefficient), followed by detail coefficients at 5 scale levels from high to low as D_5_, D_4_, D_3_, D_2_, and D_1_. Within each scale level, the coefficients *d_j_^i^* are arranged in a time-ascending order. The x-axis represents the global coefficient index.

WT coefficients as features extracted from training voxels are shown in Figure 5. A box plot in Figure 5 shows scattering ranges of WT coefficients from each scale level (*J*) grouped by a rectangle. Note that variation of the coefficient values increases with the scale level *J*, and also early-time coefficients (smaller index) within the same scale level generally show larger variation. Table 2 shows the coefficients in Figure 5 selected by the filter-based feature selection but 3 different search methods. Four coefficients with indices #1, #5, #9, and #18 were selected by all search methods, whereas the *Genetic* search picked up 2 additional coefficients. Note that the 4 common coefficients were the early-time coefficients within the scale levels where they were computed, and most of the selected coefficients were from higher scale levels (corresponding to low WT frequency components) where global shape information of the curve is captured. In addition, the early-time coefficients in each scale level were computed from early enhancement phases. Therefore, the results suggest that the global shape of the curve and intensities from early enhancement phases provide more useful information for the voxel classification and analysis of DCE-MRI.

**Table 2.** Feature Selection Results from WT Features by *Filter* Method Using Different Search Methods

Search Method	Index of Selected Coefficients
Best-first	1, 5, 9, 18
Genetic	1, 3, 4, 5, 9, 18
Greedy	1, 5, 9, 18

### PCA Feature Selection

**Figure 6.**
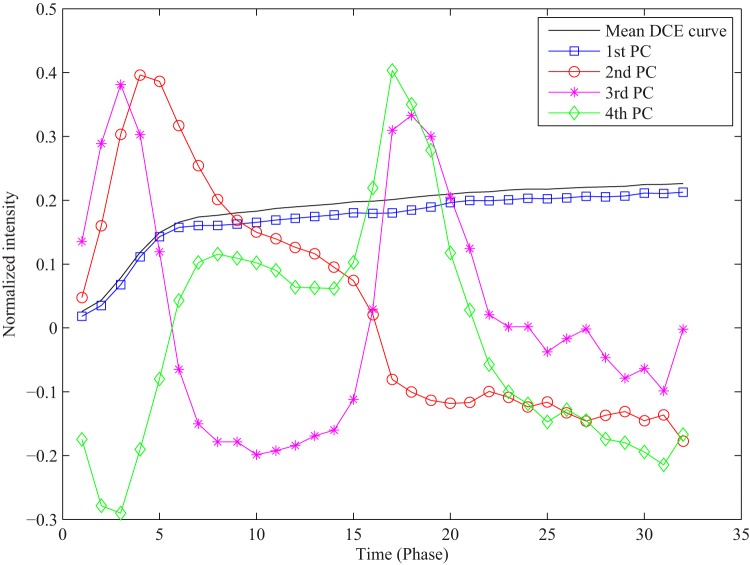
A mean DCE curve of training curves and the first 4 PCs derived from the training data set.

PCA in our application revealed that 87% of the total variance in the training data set was explained by the first PC, 92% by the first 2 PCs, and 95% by the first 4 PCs. Figure 6 illustrates the first 4 PCs with the largest eigenvalues out of 32 initial PCs. We used projection coefficients of each DCE curve to the first 4 PCs as features for voxel classification.

### Voxel Classification Results

**Figure 7.**
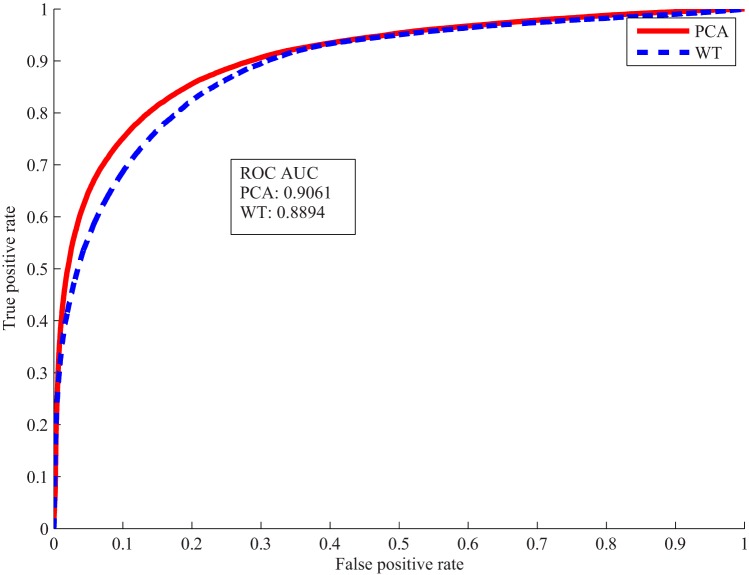
Receiver operating characteristic (ROC) curves and area under the ROC curve of voxel classification by wavelet transform (WT) and principal component analysis (PCA) features.

The selected WT features and PCA features were used to train the classifier separately. Voxel classification accuracies in Table 3 were obtained from the 2 classifiers tested on the validation data set. Both WT and PCA features achieved over 80% accuracy using only 4 coefficients. As receiver operating characteristic curves shown in Figure 7, the classifier trained on the PCA features performed slightly better than the one on the WT features.

**Table 3.** Voxel Classification on the Validation Set

Feature	Feature Dimension	Accuracy (%)
WT	4	81.2
PCA	4	83.3

Results testing the classifiers on the 6 new patients' data are shown in Table 4. The size of s-LBV of the patients ranged from ∼10% to 30% of the GTVs. To compute the DSC, the contiguous voxels classified as LBV but smaller than 1 cc were excluded, which was a criterion used in a clinical trial. Also, the DSC of s-LBV and the DSC of s-LBV plus s-HBV (representing the correctly classified voxels in the GTVs) were computed for the PCA classification. Overall, both classifiers achieved classification accuracy higher at the patient level than at the voxel level (shown in Table 3). In addition, DSCs suggest that the subvolumes with LBV classified by the proposed method have an excellent spatial overlap with those generated by the 2-step process via PK modeling. In the literature, a 0.7 of DSC or greater is accepted as excellent overlap between 2 segmentation results (19). Patient #5 did not have contiguous voxels with LBV greater than 1 cc for the DSC computation. Patient #6, who had the smallest s-LBV (<9% of GTVs), had the lowest DCS of 0.6 for s-LBV. Nevertheless, patient #6 had 0.94 of the DSC for LBV + HBV, the highest, suggesting the overall high rate of correctly classified voxels, and consisting of a high classification accuracy of 86% (Table 4). The small s-LBV resulted in the DSC being oversensitive to misclassification of the voxels with LBV. The DSCs calculated from the WT classifier were similar to the ones from the PCA classifier.

**Table 4.** Classification Accuracy and DSC on the 6 Patients' Data

Patients ID	Size of s-LBV in GTV (%)	Accuracy (%)		DSC (PCA)	
		Wavelet	PCA	LBV	LBV + HBV
1	21.4	88.1	87.2	0.7600	0.9070
2	20.0	88.8	88.9	0.8145	0.9295
3	28.0	83.9	83.6	0.7182	0.8690
4	31.1	80.9	81.0	0.7119	0.8383
5	17.9	85.9	84.3	NA	0.8813
6	8.7	86.0	86.3	0.6029	0.9435

### Subvolume Extraction

**Figure 8.**
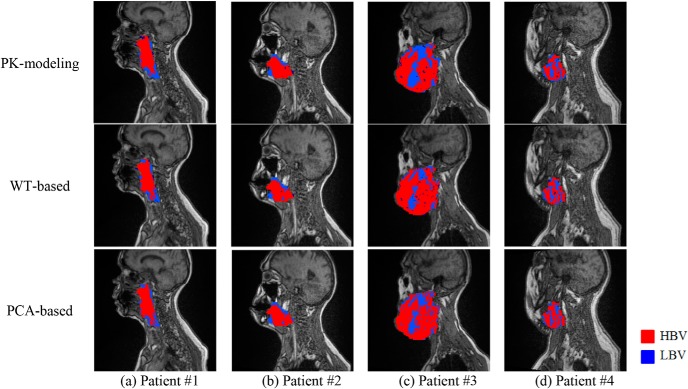
Subvolumes extracted by the 2-step method via pharmacokinetic (PK) modeling (top row), the WT-based method (middle row), and the PCA-based method (bottom row).

Figure 8 shows representative examples of subvolumes with LBV extracted by our proposed methods and the 2-step method (via PK modeling) from the first 4 patients in Table 4. Note that locations of the subvolumes with LBV mapped by the proposed and PK-based methods are corresponding well, but the boundaries of the subvolumes vary between the 2 approaches. The boundary mismatching is a typical behavior of a binary classification on continuous changes of data. For instance, LBV voxel distributions in patients #3 and #4 were more complicated compared with those in patients #1 and #2, resulting in relatively lower voxel classification accuracies and DSCs. In addition, the s-LBVs derived by the proposed methods were smaller than those by PK modeling, especially in patient #3.

### Robustness of the Classification Model to Magnetic Resonance Acquisition Protocol

It is important to understand whether our proposed machine learning model is applicable to data sets obtained by different acquisition settings or scanners without retraining the model. We conducted a preliminary test by directly applying our trained model to the DCE data acquired by a completely different protocol on a different vendor scanner in 2 patients. The acquisition parameters used for model training and for additional testing are listed in Table 5 for comparison.

**Table 5.** Comparison of Acquisition Parameters and Scanner Type Between 2 Scanners

Acquisition Parameters	Scanner 1 (Training Data)	Scanner 2 (Additional Testing)
Voxel size (mm^3^)	1.56 × 1.56 × 1.5	2 × 2 × 2
TE/TR (ms)	0.9/2.58	1.16/5.14
Temporal resolution (s)	∼3 (60 dynamic image volumes)	∼7.7 (∼32 dynamic image volumes)
Purse sequence	TWIST	Gradient Echo
Scanner	Skyra 3T, Siemens	Intera Achieva 3T, Philips

**Figure 9.**
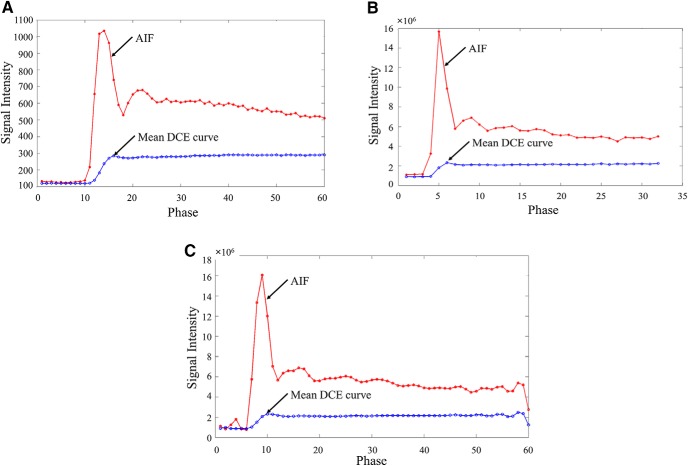
DCE curves (arterial input function [AIF] and mean curves of tumor voxels in gross tumor volumes [GTVs]) from different scanners and resampled curves. Scanner 1 (A). Scanner 2 (B). Resampled data of the curves in (B) (C).

Figure 9 shows AIFs and mean DCE curves from 2 patients' data, each scanned by the acquisition parameters in Table 5. Major differences between the 2 included the following:

(1) The DCE data in Figure 9B had only 32 dynamic image volumes (phases) with a temporal resolution of ∼7.7 seconds.
(2) The signal intensity in Figure 9B was significantly greater than that in Figure 9A by ∼104 times.

For the DCE data obtained by *Scanner 2*, we performed only additional resampling of the DCE curves (Figure 9C) to have ∼60 time points with a temporal resolution of ∼4.1 seconds, closer to 3.0 seconds in training data on *Scanner 1*. Evaluation results are shown in Table 6. The PCA-based method achieved >80% of accuracy for both patients, comparable with those in Table 4. The WT-based method had 82% and 75% of accuracy for the respective first and second patients, respectively, relatively lower than those in Table 4. In addition, all the DSCs were >0.7. The results suggest that our proposed machine learning approach is applicable to data sets obtained by different acquisition parameters and scanners with minor additional preprocessing, but no major changes in the model, to produce sufficient accuracy in the subvolume maps.

**Table 6.** Classification Accuracy and DSC on Patients' Data Obtained by *Scanner 2*

Patients ID	Size of s-LBV in GTV (%)	Accuracy (%)		DSC (PCA)	
		Wavelet	PCA	LBV	LBV + HBV
1	52.5	82.3	80.1	0.7230	0.7548
2	47.7	75.2	82.5	0.7153	0.7829

### Sensitivity of the Classification Model to the AIF Parameters

The peak and onset times of the AIF can be susceptible to errors because of numerous factors. The errors can cause variations in the normalized curves, and subsequently, affect the voxel classification and subvolume extraction. To examine the impact of the variations, we performed a classification test by varying *AIF*_*max*_ and *AIF*_*onset*_ values used in the DCE curve normalization. We simulated probable measurement variations in the *AIF*_*onset*_ by shifting the onset time by ±2 and ±1 time points from the originally identified onset time, and in the *AIF*_*max*_ by changing the peak intensity by ±10% from the original values. Considering the original onset time and peak of AIF (${\text{AIF}}_{\text{onset}}^{\text{orig}}$ and ${\text{AIF}}_{\text{max}}^{\text{orig}}$), we had 5 onset times at each *AIF*_*max*_ and 3 maximum values of AIF at each onset time for the test. We used DCE data of patients #1, #2, and #3 in Table 4 for the test.

**Figure 10.**
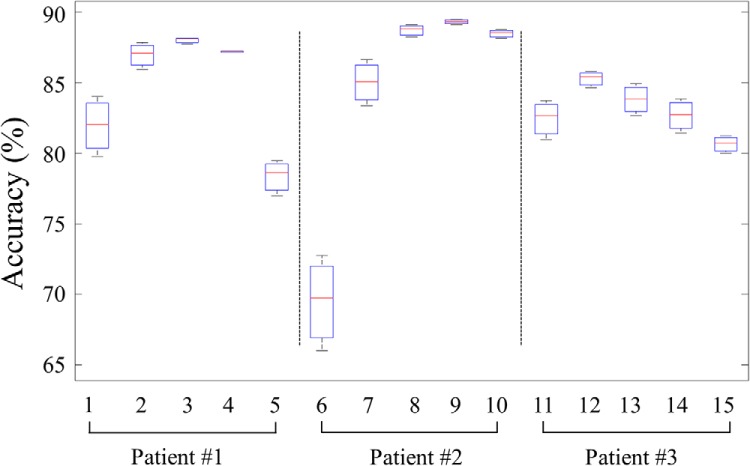
Box plot of accuracy variations at 5 different onsets of AIF when varying *AIF*_*max*_ for patients #1–3. The x-axis indexes *AIF*_*onset*_ in an ascending order (−2, –1, 0, 1, and 2) for patient #1, followed by patients #2 and #3.

Box plots of accuracy variation at each AIF_onset_, when varying AIF_max_, in the 3 patients are shown in Figure 10. Only at 2 instances, the classification accuracy was approximately <80%, for which both onset times were shifted by 2 time points from the originals. The variation in the maximum value of AIF had a lesser extent of impact on the classification accuracy than onset time. One time point shift (∼3 seconds) in the onset of AIF did not cause >4% decrease in the classification accuracy even with +10% of variation in the peak of AIF, which is consistent with the results tested on the DCE data acquired with a completely different protocol on a different scanner.

## Discussion

In this paper, we proposed a temporal feature-based machine learning approach for extracting the tumor subvolumes with LBV from DCE-MRI curves in patients with HN cancers. Our previous research shows that large subvolumes of tumors with LBV in HN cancers before RT and persisting during the early course of RT are significantly related to the tumor local and regional failure (8). In this study, we trained and validated the SVM classifier based on WT features or PCA features directly extracted from the DCE data to create the tumor subvolumes with LBV. The validation indicates that we are able to achieve fairly high voxel classification accuracy and the DSC as ∼82% and 0.72, respectively, compared with the 2-step method via PK modeling. In addition, we found that WT coefficients from higher scale levels are more informative, to yield high accuracy and the DSC, than ones from lower scale levels for our application. The former captures global shape characteristics (low-frequency components) of the curve, whereas the latter captures high-frequency fluctuations. Furthermore, the proposed machine learning method has a fair amount of tolerance to the DCE data acquired by different protocols on different scanners and the AIF onset and peak. The method developed in this study can be fully automated and is scalable for extracting the significant tumor subvolume in HN cancer, which could be an important tool to support future clinical trials that adapt individual treatments based on the response of this subvolume to therapy.

Discrete WT has been applied similarly to other studies to extract temporal features of the DCE curves for voxel cluster analysis (20, 21). In these studies, the WT is basically the same as ours, except for the mother WTs (Ψ), but WT coefficient selections differ from ours. In Whitcher et al.'s study (20), the approximation coefficient (A_J_) and detail coefficients in D_1_ were discarded. However, the approximation coefficient was found to be the most informative in our study. In addition, the coefficients in higher scale levels (J > 1) were all selected in Whitcher et al.'s study (20), whereas only several of them were found to be useful in our application. WT shrinkage that sets small coefficients below a threshold to zero was used by Li et al. (21) for feature selection. However, the wavelet shrinkage is not suitable for our classification-based methodology, as the number of selected coefficients varies by the threshold.

Our proposed approach has several advantages over other approaches for tumor region segmentation using features from DCE curves. The most promising advantage could be that the resulting subvolumes are similar to ones defined by physiological parameters. Basically, our approach is also a method for segmenting a heterogeneous tumor volume into subregions (subvolumes in this paper) based upon the image features of interest. However, ours is different from others, in that its resulting subregions are already associated with physiological characteristics of tumors (ie, BV) and even cancer treatment outcomes, whereas those from others are generally not. Subregions (or clusters) in other approaches may be well characterized by the imaging features, but additional tests are required to associate them with tumor behavior or underlying structure of the tissue (11, 15). In our approach, voxels are characterized by not only (explicitly) features extracted from the DCE curves as in other approaches but also (implicitly) physiological parameters obtained from PK model fitting. The physiological parameters, however, are not directly included in the features used to train the model. Our model enables to apply to new patients' data in the usage phase without any computation of physiological parameters and obtain subvolumes similar to physiological parameter-derived ones. Therefore, our approach is attractive by taking advantages of the PK model-free approach, but yielding the results similar to the PK model-based method, which could facilitate the development of the real-time decision-making supportive tools in diagnosis and therapy assessment. Another advantage of our method is the simplicity of the WT-based feature extraction, which requires only simple sum and difference computations of pairs of floating numbers. In addition, the multiresolution analysis of WT, decomposing a signal into different frequency components, can provide richer choices for feature selection.

Preprocessing of the DCE curves is generally required by quantification methods. Our approach needs substantially less computation efforts than others, for example, using a gamma variate function to fit the DCE curves to compute perfusion-related parameters, such as peak enhancement, time-to-peak, and so on. However, the fitting could be another source of errors, as it can be frequently failed with noisy signals, and the fitting results are greatly susceptible to initialization of fitting parameters.

It is worthwhile to mention another potential benefit of our approach for the DCE-MRI analysis. Our experimental results revealed that, in WT features, low-frequency components are more informative than higher-frequency components. Therefore, we may be able to further decrease the temporal resolution of the DCE data from ∼3 seconds (currently used) and thereby increase the spatial resolution of the dynamic images to more accurately delineate the tumor heterogeneity. Decreasing the temporal resolution could challenge the results from conventional PK modeling, where a higher temporal resolution is desirable for more accurate DCE-MRI parameter estimation (1). A high temporal resolution is often a tradeoff spatial resolution. Our method could potentially overcome this disadvantage of the PK model.

We ultimately aim to develop an automated, fast, and generalizable model for DCE-MRI quantification. Our current model uses 2 parameters, namely, AIF_max_ and AIF_onset_, which were manually obtained and used for preprocessing of the DCE curves; however, all other processes in our framework are fully automated. Compared with other PK model-free approaches, our method does not fit the DCE curves to extract the *ad hoc* empirical parameters, namely, the first contrast uptake, time-to-peak, peak enhancement, and so on (in general, called semiquantitative parameters). However, such critical information is extracted by WT coefficients and ready for classification. Therefore, our approach could be generalized for quantification of DCE-MRI data in other cancers or acquired by different image protocols with minimum or no modification.

## Conclusion

In this paper, we propose a framework of temporal-feature extraction machine learning for analyzing the DCE-MRI data to detect tumor subvolumes with LBV, which have been shown to be significantly associated with local and regional failure in HN cancer after CRT. Further investigation using a larger data set with clinical endpoints obtained by various acquisition protocols is necessary to improve the performance, find the relationship between changes in the subvolumes of LBV and treatment outcomes, and extend the framework to other types of tumors.
